# Obituary

**Published:** 1910-02-19

**Authors:** 


### Obituary.

The death is announced of Mr. William Herbert Hillyer, who had studied at both Edinburgh and Glasgow, and took his diploma at Edinburgh University in 1887, becoming L.F.P.S. of Glasgow in the same year. He then took up his residence in London.

The death is announced at the age of 67 of Dr. Thomas Kilner Clarke, J.P., one of the oldest and most prominent surgeons in Huddersfield. He took his M.A. at Cambridge in 1871; M.D. (Trinity College, Dublin) in 1873; F.R.C.S. in 1872; was ex-President of the Yorkshire British Medical Association, formerly surgeon to the Huddersfield Infirmary, member of various societies, and a frequent contributor to the medical journals.

The death is reported at Kibworth, Leicestershire, of Sir Charles Hayes Marriott, F.R.C.S., at the age of 76 Sir Charles Marriott's Leicestershire family had for generations been associated with medicine and surgery. He was educated at University College, London. Soon after beginning practice he was appointed house surgeon to the Leicester Infirmary, and up to the date of his retirement he continued to be associated with the infirmary in an honorary capacity as operating surgeon. In the field of private practice he rapidly became prominent, his great skill in operations in the early days of anæsthetics gaining for him a reputation that was by no means local. He took an active interest in the development of aseptic and anti-septic surgery, foreseeing their remarkable effect in the alleviation of suffering and in the direction of a diminished mortality. In later years, while retaining his high reputation in the operating theatre, he was much sought after as a consulting physician. For a period of over 40 years he gave his services to the Leicester Infirmary, and his retirement from the profession a few years ago, owing to failing health, was made the occasion of a public recognition of his benevolent work. He was one of the founders of the Leicestershire Nursing Home, was a Justice of the Peace and a Deputy-Lieutenant of Leicestershire, and in 1904 the King conferred upon him the honour of knighthood. He married in 1864 a daughter of the late Rev. John Gibson, and leaves four sons.

